# Supplementary material for: A verifiably secure and robust authentication protocol for synergistically-assisted IoD deployment drones
Source: PLoS One. 2025 Mar 10;20(3):e0314475. doi: 10.1371/journal.pone.0314475 (PMC11892824; doi:10.1371/journal.pone.0314475)
Supplement: Attachment — Submitted filename: Rebuttal Letter.docx [file pone.0314475.s002.docx]

Subject:	***REBUTTAL LETTER***

Memo;

We are presenting a rebuttal letter that demonstrates the answer to the reviewers’/Editor’s concerns and the changes made in the article. We have taken great care to ensure that our explanations for each concern are clear and comprehensive. These concerns have been incorporated in the revised and modified version of the article, and our responses to each one are explained as follows. We hope the Editor and reviewers will accept our efforts.

**EDITOR’S COMMENTS**

**Comment 1:** We note that Figure 1 in your submission contains copyrighted images.

**Authors’ Response:** Thanks Supporting Information **S1 Fig. Proposed System Model.** (.pdf)

**Authors’ Action:** The System Model diagram represented in Fig. 1 consisted of three entities:

***DRONE OF TYPE 1*** have been taken from (<https://ieeexplore.ieee.org/document/9530542>)

***DRONE OF TYPE 2*** has been taken from ([https://ieeexplore.ieee.org/document/9530542](https://vajiramandravi.com/upsc-daily-current-affairs/prelims-pointers/what-is-switchblade-600/))

**Ground Control Station** symbol has been taken from (<https://ieeexplore.ieee.org/document/10480416>) We also has cited the Proposed model from the above articles.

**Comment 2:** While the paper presents a potentially valuable contribution, I believe it requires significant revisions to reach a publishable standard.

**Authors’ Response:** Thanks

**Authors’ Action:** The whole article is modified

**Comment 3:** Improve the structure and clarity of the introduction, clearly outlining the problem statement and objectives.

**Authors’ Response:** Thanks for pointing out such a technical mistake

**Authors’ Action:** The introduction section is revised by including problem statement, motivation and objectives of the research work.

**Comment 4:** Update the literature review with recent references and critically analyze existing protocols.

**Authors’ Response:** Thanks

**Authors’ Action:** We have also updated the literature review section by adding more articles from the existing literature and analyzed them critically

**REVIEWER 1 COMMENTS**

**Comment 1:** The abstract needs to be revised a bit and stressing more on specific key contribution and originality. Try reducing general knowledge aiming to attract the reader to select the paper to study and refer to as well as get motivated toward the work to continue research in similar direction. Try reducing answering ‘how’ question within the abstract, i.e. making it focus on ‘what’ question as its main phrasing presentation?

**Authors’ Response:** Thanks

**Authors’ Action:** The whole abstract is revised

**Comment 2:** Give more elaboration on the real need for utilizing this elliptic curve crypto authentication proposal among other approaches. What is wrong in the normal other related hash authentication methods requiring this kind of complex research. Try to support your explanation via real-life examples.

**Authors’ Response:** Thanks

**Authors’ Action:** Yes, this concern has been addressed in both introduction section as well as related works section.

**Comment 3:** The IoD security presented is very promising showing its limited resources trade-off to security and robustness improvement as challenging processes. Try to benefit from the light crypto and authentication secrecy researches below linking to the secrecy vs cost complexity attempts provided:

== "Enhancing Speed of SIMON: A Light-Weight-Cryptographic Algorithm for IoT Applications", Multimedia Tools and Applications (MTAP) 78:32633–32657 (2019)

== "Simulating Light-Weight-Cryptography Implementation for IoT Healthcare Data Security Applications", International Journal of E-Health and Medical Communications (IJEHMC) 10(4):1-15 (2019)

== "Integrating Light-Weight Cryptography with Diacritics Arabic Text Steganography Improved for Practical Security Applications", Journal of Information Security and Cybercrimes Research (JISCR) 3(1):13-30 (2020)

== "Secure Mobile Computing Authentication Utilizing Hash, Cryptography and Steganography Combination", Journal of Information Security and Cybercrimes Research (JISCR) 2(1):73-82 (2019)

== "Engineering Graphical Captcha and AES Crypto Hash Functions for Secure Online Authentication", Journal of Engineering Research (JER) 11(3): 100-111 (2023)

== "Watermarking Images via Counting-Based Secret Sharing for Lightweight Semi-Complete Authentication",  International Journal of Information Security and Privacy (IJISP) 16(1): 1-18 (2022)

== “Adjusting counting-based secret-sharing via personalized passwords and email-authentic reliability”, Journal of Engineering Research (JER), 12(1): 107-121 (2024)

**Authors’ Response:** Thanks for providing an important literature articles

**Authors’ Action:** The aforementioned articles are totally cited and taken help.

**Comment 4:** The testing needs more elaboration in different ways. You need to clarify more simple example given showing other possible scenarios with real details stressing on your proposal among others. Note that the example cases need to further consider not being system-confusion in several models as well as being in misleading technical appearance.

**Authors’ Response:** Thanks

**Authors’ Action:** The performance analysis subsection consisted of measuring communication and computation costs, then comparing with state of the art schemes in terms of both performance metrics and security functionalities. At the end, we have also showing “the percentage improvement of the proposed protocol over others” in terms of communication and computation costs.

**Comment 5:** Combining cryptography and hash is used here as very promising philosophy to stand the IoD trust authenticity privacy system against many security attacks. Although this combination is noted as extra payload in this direction, briefly link your work idea to the complexity of presented IoT similar combinations within the following studies:

== "Efficient computation of Hash Hirschberg protein alignment utilizing hyper threading multi-core sharing technology", CAAI Transactions on Intelligence Technology, IET (IEE) - Wiley, 7(2): 278–291 (2022)

== "Integrity verification for digital Holy Quran verses using cryptographic hash function and compression", Journal of King Saud University - Computer and Information Sciences 32(1):24-34 (2020)

**Authors’ Response:** Very interesting article, thank for providing such an important knowledgeable articles.

**Authors’ Action:** cited and take benefits.

**Comment 6:** The work lack reasoning within given comparisons. It needs many more logical thinking justifications supporting its contrasting to others proofing its real applicability. It also needs elaboration proofing fairness in the results and comparison, especially testing the schemes on variation of similar security IoD schemes. It needs some indication with more circumstances of these results. Explain more the observations feedback of the proposed work vs. others ?

**Authors’ Response:** Thanks

**Authors’ Action:** Yes, more explanation about comparison has been given in the last section before conclusion.

**Comment 7:** This work is elliptic curve crypto procedure for its less computation high resilience security. Remark on the proposal given in light of considering the following ECC encryption confidentiality attempts:

== “Fast 160-Bits GF(p) Elliptic Curve Crypto Hardware of High-Radix Scalable Multipliers”, International Arab Journal of Information Technology (IAJIT) 3(4):342-349

== “Area Flexible GF(2k) Elliptic Curve Cryptography Coprocessor”, International Arab Journal of Information Technology (IAJIT) 4(1):1-10

== "Implementation of a pipelined modular multiplier architecture for GF(p) elliptic curve cryptography computation", Kuwait Journal of Science and Engineering (KJSE) 38(2B):125-153

== "Enhancing Medical Data Security via Combining Elliptic Curve Cryptography with 1-LSB and 2-LSB Image Steganography ",International Journal of Computer Science and Network Security (IJCSNS) 20(12):232-241 (2020)

**Authors’ Response:** Thanks

**Authors’ Action:** Very interesting work, we have cited in our article.

**Comment 8:** The list shown in Table 6 of percentage improvement linking proposed scheme over state-of-the-art schemes as well as comparative analysis of security functionalities of Table 7 for the proposed scheme is kind of misleading as reported needing to justify on similar testing platform and configuration circumstances. It is recommended that these tables be elaborated and justified fair to be acceptable logical attitude.

**Authors’ Response:** Thanks

**Authors’ Action:** revised, elaborated, and given a full attention to the last section.

**Comment 9:** Conclusion needs reconsideration. It needs to highlight the research main contribution with some brief indications and numerical improvement percentages to keep with the reader. Also, the conclusion needs to present some more ideas of open research and future work for researchers to build upon for further advancements.

**Authors’ Response:** Thanks

**Authors’ Action:** Conclusion is also revised.

**REVIEWER 2 COMMENTS**

**Comment (1):** The names of variables and functions should be in italics.

**Authors’ Response:** Thanks

**Authors’ Action:** Revised in the modified version of the article

**Comment (2):** What is the difference between the contents in Table 2 and Table 3? Why not merge?

**Authors’ Response:** Thanks

**Authors’ Action:** Actually, the notations used by [35] are represented in Table 2, while the notation used for designing the proposed scheme is represented in Table 3. Both are separate sections, so we have keep for reader understandability about [35] and proposed.

**Comment (3):** The experimental section suggests adding some bar charts and graphs to compare the cost of different schemes.

**Authors’ Response:** Thanks

**Authors’ Action:** More details have been added

**Comment (4):** The discussion or comparisons with more recent related schemes, such as lightweight trustworthy message exchange in unmanned aerial vehicle networks, icra: an intelligent clustering routing approach for uav ad hoc networks, pbag: a privacy-preserving blockchain-based authentication protocol with global-updated commitment in iovs, instead of conventional schemes are suggested.

**Authors’ Response:** Thanks

**Authors’ Action:** The proposed work is also compared with the mentioned article in terms of both security and performance.

**Comment (5):** The writing of the paper needs further improvement.

**Authors’ Response:** Thanks

**Authors’ Action:** The whole article revised from scratch

Dr. Ali Algarni

[al.alqarni@ub.edu.sa](mailto:al.alqarni@ub.edu.sa)
